# Differential Effects of Soy Isoflavones on the Biophysical Properties of Model Membranes

**DOI:** 10.1021/acs.jpcb.3c08390

**Published:** 2024-02-28

**Authors:** Jamie Gudyka, Jasmin Ceja-Vega, Katherine Ivanchenko, Wilber Perla, Christopher Poust, Alondra Gamez Hernandez, Colleen Clarke, Shakinah Silverberg, Escarlin Perez, Sunghee Lee

**Affiliations:** Department of Chemistry and Biochemistry, Iona University, 715 North Avenue, New Rochelle, New York 10801, United States

## Abstract

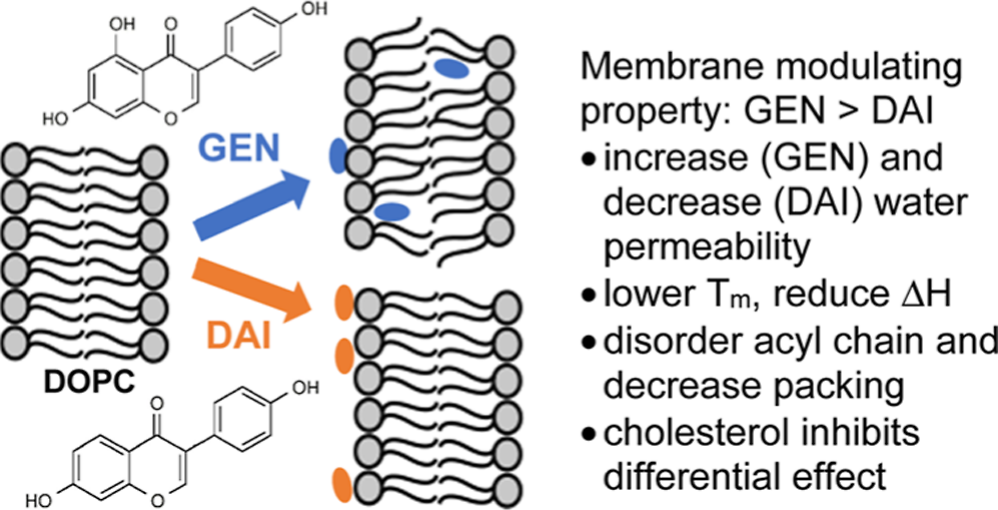

The effects that the main soy isoflavones, genistein and daidzein, have upon the biophysical properties of a model lipid bilayer composed of 1,2-dioleoyl-*sn*-glycero-3-phosphocholine (DOPC) or DOPC with cholesterol (4 to 1 mol ratio) have been investigated by transbilayer water permeability, differential scanning calorimetry, and confocal Raman microspectroscopy. Genistein is found to increase water permeability, decrease phase transition temperature, reduce enthalpy of transition, and induce packing disorder in the DOPC membrane with an increasing concentration. On the contrary, daidzein decreases water permeability and shows negligible impact on thermodynamic parameters and packing disorder at comparable concentrations. For a cholesterol-containing DOPC bilayer, both genistein and daidzein exhibit an overall less pronounced effect on transbilayer water permeability. Their respective differential abilities to modify the physical and structural properties of biomembranes with varying lipid compositions signify a complex and sensitive nature to isoflavone interactions, which depends on the initial state of bilayer packing and the differences in the molecular structures of these soy isoflavones, and provide insights in understanding the interactions of these molecules with cellular membranes.

## Introduction

Genistein (GEN) and daidzein (DAI) are natural compounds distributed in *Leguminosae* species and are consumed through the human diet including from soy and soy-derived food products.^1,2^ They are polyphenolic compounds and are the two major isoflavones of soybeans. Due to their structural similarity to estradiol, natural estrogen, and their affinity for binding estrogen receptors (ERs), they are classified as phytoestrogens and are said to exert estrogenic effects on human beings.^3−5^ Soy isoflavones have been shown to have numerous potential beneficial effects on human health, such as antibacterial, antiviral, anti-inflammatory, antioxidant, and antitumor properties, as well as the ability to induce hormonal and metabolic changes.^6−9^ These properties have been suggested to play a preventive role in age-related diseases including cardiovascular disease, cancer, reproductive disorders, osteoporosis, and neurodegenerative diseases.^3,10,11^ This has led to numerous clinical trials for pharmacological efficacy in the treatment of these various disorders. In addition to the ability to interact with ERs, the biological activities of soy isoflavones have other ER-independent signaling mechanisms including protein kinase regulation, enzyme inhibition in steroid biosynthesis, the ability to reduce oxidative damage, influence the immune reaction, cell cycle control and metastasis inhibition, induce apoptosis, and target critical oncogenic signaling pathways.^5,12−14^ It has also been reported that isoflavones can alter the physical properties of the bilayer component of the membrane into which proteins are embedded in a nonspecific manner, resulting in the modulation of the function of diverse integral membrane proteins and membrane-dependent processes.^15,16^ This bilayer-mediated mechanism often interferes with the identification of a specific molecular target and adds additional challenges in assessing bioefficacy and understanding the molecular mechanism of action.^17,18^

Many previous studies have shown that GEN and DAI interact with cell membranes^19^ and influence cell mechanical properties, including membrane tension and fluidity.^20^ Despite previous studies on nonspecific interactions between isoflavones and lipid bilayer membranes, conducted *via* diverse experimental and computational methods, there are numerous reports regarding their positioning within the membrane bilayer and their potency in modifying the physical and structural properties of membranes, some of which contradict others. Table 1 shows some examples of these studies for a wide range of lipid compositions.

**Table 1:** Examples of the Effect of GEN and DAI on the Lipid Membranes with Various Lipid Compositions

isoflavones	lipid composition	characterization	impact on membrane	ref.
GEN	DOPC, DPhPC	X-ray scattering, MD simulation	decreases bilayer thickness and area compressibility modulus; softens bilayer; orients parallel to the bilayer surface	(21)
	erythrocyte	EPR spectroscopy	decreases fluidity; locates near the hydrophilic surface	(22)
	DPhPC, DOPC	gA channel assay	increases the lifetime of gramicidin A channel (gA); alters bilayer elastic properties	(23)
	SLPC	fluorescence polarization	decreases fluidity; partitions into the hydrophobic cores	(24)
	DMPC, DMPS, eggPC	turbidity, DSC	does not aggregate liposomes	(25)
	DPPC	FT-IR, ^1^H NMR, EPR	decreases fluidity; locates in the lipid/water interface	(26)
	DPPC	ATR-IR	increases fluidity (loosens the packing of molecules and enhances gauche conformers)	(27)
	DMPC	FT-IR, ^1^H, ^31^P NMR, DSC	decreases fluidity, increases packing, and restricts the motion of choline	(28)
	DMPC, DPPC	fluorescence, ESR spectroscopy	locates dominantly in the lipid headgroup	(29)
	POPC/Chol (20 mol %)	fluorescence polarization	decreases fluidity, hydrophobic region of the membrane lipid bilayer	(30)
DAI	DOPC, DPhPC	X-ray scattering, MD simulation	decreases bilayer thickness and area compressibility modulus (less than GEN); softens bilayer; orients parallel to the bilayer surface	(21)
	erythrocyte	EPR spectroscopy	increases fluidity; locates in deeper regions of the membrane	(22)
	DPhPC, DOPC	gA channel assay	little effect on gA lifetime	(23)
	DPPC/DPPG	fluorescence spectroscopy	increases fluidity	(31)
	DMPC, DMPS, eggPC	turbidity, DSC	aggregates liposomes; locates in the headgroup/interfacial region	(25)
	soybean PC	fluorescence polarization	decreases fluidity; locates at the membrane surface	(32)
	POPC/Chol (20 mol %)	fluorescence polarization	no effect	(30)

Contrasting effects of these structurally similar soy isoflavone molecules, GEN and DAI (see Table 2 for molecular structures), upon interaction with model and biological cell membranes have been reported. For example, DAI, but not GEN, was reported to bind to large unilamellar vesicles and induce the aggregation of liposomes.^25^ Using electron paramagnetic resonance spectroscopy, it has been reported that GEN and DAI have disparate effects on erythrocyte membrane fluidity: GEN reduced erythrocyte membrane fluidity near the hydrophilic surface, whereas DAI at the same concentration increased the fluidity of deeper layers of the erythrocyte membrane.^22^ Using fluorescence polarization, GEN was shown to rigidify the model tumor cell membranes in the hydrophobic regions of membrane lipid bilayers, while DAI was not effective at the same concentration.^30^ It was also reported that GEN (but not DAI) reduced the membrane fluidity of human colon tumor cells.^33^ In addition, GEN was shown to have greater ability to suppress metastatic prostate cancer cells *via* decreasing membrane fluidity, but not DAI, at the same concentration.^34^ In a study of the flavonoids’ effect on intestinal integrity using the *in vitro* Caco-2 cell model, DAI and GEN showed a disparate effect on intestinal barrier integrity, where DAI was reported to enhance the basal tight junction (TJ) integrity in intestinal cells and GEN presented protective effects on the TJ integrity against harmful substances.^35^ Using the gramicidin A (gA) channel, it was demonstrated that GEN has significant effects on gA channel function by increasing gA ion-channel lifetimes and the appearance rate in planar phospholipid bilayers, but DAI has little effect on gA channel function.^23^

**Table 2:**
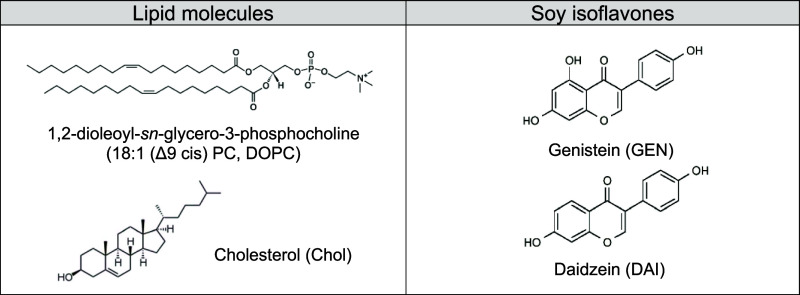
Structures of DOPC, Cholesterol, and Isoflavone Molecules

One can discern that many of the results in Table 1 are not mutually consistent, notably those having to do with fluidity, whereby some assays indicate increasing fluidity for GEN and DAI and some indicate the opposite. It should be noted that if, for example, fluorescence polarization is employed to assay a given flavonoid’s impact on the membrane, this necessitates the use of a probe molecule, which itself may act as a membrane perturbant as does the flavonoid. Furthermore, different techniques may not all measure the same properties of a membrane (*e.g.*, some may be local properties and some global). It is noted that similar difficulties have been reported when using nine different methodologies to determine membrane order in serotonin-perturbed membranes: the results were found to lack mutual consistency.^36^ Regardless of the intrinsic difficulties attendant to the interpretation of different fluidity assays, however, the field of membrane–flavonoid interactions is in need of clarification.

**Figure 1:**
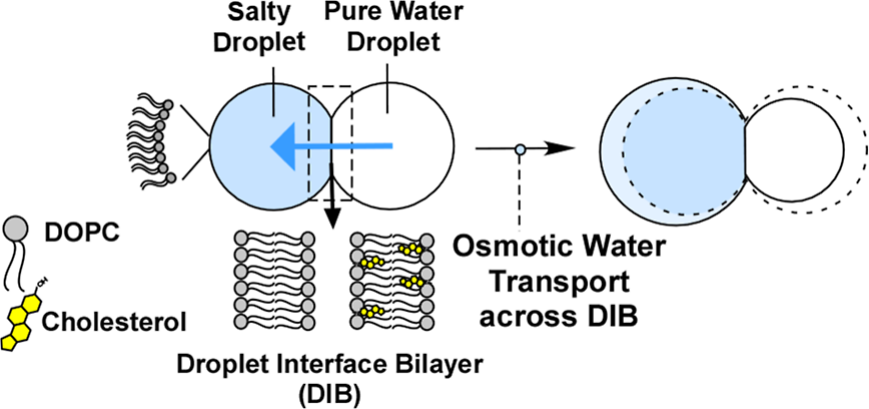
General schematic of the DIB-based osmotic water permeability experiment. DIB is formed into a membrane-mimetic structure. In this study, bilayers are composed of DOPC and DOPC/Chol (4/1 mol/mol). When an osmotic pressure imbalance exists between two adjoining aqueous microdroplets in a DIB, water transport occurs through the DIB, leading to a measurable change in the droplet diameter.

Cell membranes play pivotal roles in a wide array of biological processes.^37^ They are responsible for establishing and maintaining transmembrane gradients, compartmentalizing cells, facilitating inter- and intracellular communication, enabling cell–cell recognition, and orchestrating energy transduction events. At the heart of cell membranes lies the lipid bilayer, which serves as the fundamental structural framework. This lipid bilayer acts as a selectively permeable barrier, separating the intracellular and extracellular environments. The transport of small molecules through the lipid bilayer is crucial for the fundamental functions of natural cellular systems. Specifically, the passive movement of water molecules across cellular membranes plays a vital role in maintaining the organism’s homeostasis. The efficiency of such transport depends on various factors, with the membrane’s structure and its constituent lipids being particularly influential. Since the water transport process is intricately linked to the inherent structure of the lipid bilayer, elucidating the dynamics of water permeation through bilayers has the potential to unveil crucial insights into the bilayer’s structural characteristics.^38^ In our previous research studies, we developed a methodology to examine the barrier functionality of lipid bilayers against water permeation, employing a model membrane formed by the droplet interface bilayer (DIB), as shown in Figure 1.^39,40^ A DIB is generated by bringing together aqueous microdroplets enclosed by lipid monolayers, resulting in the formation of an interfacial region with a structure closely resembling the double-leaflet lipid bilayer found in cell membranes.^41,42^ When an osmotic pressure imbalance exists between two adjoining aqueous microdroplets in a DIB, water transport occurs through the DIB, leading to a measurable change in droplet diameter, as schematically depicted in Figure 1 with a blue arrow indicating the direction of water movement. Through the use of water as a molecular probe, we illustrated that the rate at which water traverses a bilayer is highly responsive to the physical state of cell membranes. Consequently, delving into water permeability studies can significantly enrich our comprehension of the intrinsic barrier properties inherent in membrane architecture.^43−46^ In recent studies, we have also investigated the effects of a wide range of biologically important molecules on membrane permeability and, in turn, membrane structure.^47−50^ Our previous studies have shown the intriguing ability of bioactive molecules and phytochemicals to sensitively and variously respond to model membranes of diverse compositions in modulating physical properties such as transbilayer water permeability and thermodynamic, structural, and surface properties.

Flavonoids are known to induce changes in the barrier functions of lipid membranes. For example, it has been reported that flavonoid incorporation into vesicle membranes causes stress in the DPPC bilayer packing and results in changed barrier functions, as evidenced by temporal changes in flavonoid concentration in the aqueous medium surrounding liposomes.^51^ Based on a calcein release study, it was demonstrated that flavonoids increase the membrane permeability of egg phosphatidylcholine (EPC) vesicles and that the more hydrophobic flavonoids exhibit a greater tendency to increase membrane permeability.^52^ In addition, GEN and its derivatives are shown to increase the permeability of EPC liposome membranes, as studied by a calcein-leakage assay, indicating that these molecules can decrease membrane integrity and induce bilayer destabilization depending on molecular structures.^53^ However, there are no reported studies of the ability of GEN and DAI to modulate the membrane barrier function during water permeation.

In this study, we compare the interactions of GEN and DAI with 1,2-dioleoyl-*sn*-glycero-3-phosphocholine (DOPC) biomembranes in the presence and absence of cholesterol to study their ability to modify the barrier function and other relevant physical properties of the membrane. As one of the most important components of animal cell membranes, cholesterol comprises 20–50 mol % of total lipid in the plasma membrane.^54^ Its major role in the membrane is considered to be its ability to modulate the structural and physicochemical properties of the plasma membrane lipid bilayer and regulate the function of a wide range of *trans*-membrane proteins.^54,55^ The metabolism and concentration of cholesterol are known to be altered in cancer cells depending on the type of cancer and its stage,^56,57^ and each is correlated with cancer progression and immune responses.^58,59^ In this work, two DIB-based model membranes consisting of pure DOPC and DOPC with cholesterol (4 to 1 mol ratio) are constructed, and transbilayer osmotic water transport parameters for each membrane composition are determined as a function of GEN and DAI concentrations. In addition, differential scanning calorimetry (DSC) and confocal Raman microspectroscopy are used to monitor changes in the thermotropic, structural, and packing properties of membranes as a function of isoflavone molecule concentrations.

## Materials and Methods

### Sample Preparation

Structures of DOPC, cholesterol (Chol), and the two studied isoflavone molecules are shown in Table 2. GEN has three hydroxyl substituents and DAI has two, structurally differing by one −OH group in the polyphenolic ring. DOPC was obtained from Avanti Polar Lipids, Inc. (Alabaster, AL) with 99+ % purity and stored at −20 °C. GEN and DAI (each >99%) were purchased from LC Laboratories (Woburn, MA) and stored at −20 °C. Chol and squalene (2,6,10,15,19,23-hexamethyl-2,6,10,14,18,22-tetracosahexaene; C_30_H_50_; SqE) of the highest purity available were purchased from Sigma-Aldrich and stored at reduced temperatures (−20 °C for Chol and 2–8 °C for SqE). In order to avoid photooxidation of lipids and isoflavone molecules, all samples were prepared in an amber bottle or a bottle wrapped with aluminum foil. All reagents were freshly prepared immediately before use in experiments.

In order to prepare a squalene solution containing DOPC (with or without Chol), a chloroform solution of DOPC was evaporated under inert gas to make a dried thin film of lipid (or lipid mixture), followed by overnight vacuum drying for complete removal of any residual solvent. GEN and DAI [stock solution is prepared using chloroform/methanol (8/2, v/v) as solvents] were codissolved with the lipid or lipid mixture in an appropriate mol ratio, followed by the complete evaporation of all solvents to generate a dried isoflavone/lipid film of a defined mol ratio. For water permeability experiments, this dried lipid film was dissolved in SqE to a total lipid concentration of 5 mg/mL. For the DOPC sample containing Chol, a 4 to 1 mol ratio of DOPC/Chol mixtures was used. The experiments were performed using unbuffered aqueous solutions at pH 6–7. For DSC experiments, the dried isoflavone/lipid films described above were subsequently rehydrated with pure water to a total lipid concentration of ∼16 mg/mL and vortexed vigorously for about 5 min to obtain a suspension of multilamellar vesicles (MLVs), followed by bath sonication for ca. 30 min. For the Raman microspectroscopy experiment, the generated MLVs were further treated by seven cycles of freeze–thaw using liquid nitrogen. Osmotic solutions were prepared by dissolving NaCl, nominally at a concentration of 0.1 M, in purified and deionized water with a high resistivity of 18.2 MΩ·cm, utilizing a Millipore water purification system (Direct Q-3). The osmolality (in mOsm/kg) of all solutions was measured by a vapor pressure osmometer (VAPRO model 5600). All solutions were freshly prepared each time prior to use.

### Water Permeability Evaluation Using the Droplet Interface Bilayer

The evaluation of water permeability was performed using a model membrane formed by the DIB method (Figure 1). Additional details on the determination of water permeability are described in Supporting Information. Our experimental setup and procedure for water permeability measurement using the DIB method have been described in previous papers, and a similar setup was used for this experiment.^43^ A complete setup includes a micropipette manipulation station built on an inverted microscope, with a camera directly attached to the microscope for real-time video recording of the microdroplets and their size changes. A pair of osmotically unbalanced aqueous droplets is created in an immiscible solvent (SqE), which contains lipids that include GEN or DAI at a given molar ratio. All water permeability experiments were carried out at 30 °C using a custom-built temperature-controlled microchamber, which was thermostated *via* an external circulating water bath. The recorded videos were postanalyzed to measure the dimensions of droplets and contact areas using custom-built image analysis software. All droplet pairs had substantially the same initial size relative to each other in the diameter range of 100 ± 5 μm. All results are expressed as the mean ± standard error of the mean (*n* ≥ 30 for water permeability experiments).

### Thermotropic Property Measurement Using Differential Scanning Calorimetry

DSC measurements were performed on suspensions of MLVs composed of DOPC or DOPC with Chol (4 to 1 mol ratio) including concentrations of isoflavones using a TA Q2000 DSC instrument. The TA Universal Analysis software was used to determine the main phase transition temperature (*T*_m_), the temperature at the apex of the endothermic transition peak, and the phase transition enthalpy (Δ*H*), the integrated area under the heat capacity curve. About 15 μL of the MLV suspension prepared as described in the Sample Preparation section was hermetically sealed, heated, and cooled at rates of 5 °C/min from −40 to 0 °C under high-purity nitrogen with a flow rate of 50 mL/min. All experiments were repeated with three independently prepared samples, and each sample was cycled three times. Reproducible results were obtained without any hysteresis.

### Confocal Raman Microspectroscopic Measurements

The Raman spectra for supported lipid bilayers with different concentrations of isoflavone molecules were acquired by utilizing a confocal Horiba XploRA INV instrument (Nikon Eclipse Ti–U). The instrument is equipped with an internal air-cooled solid-state laser emitting at 532 nm and a thermoelectrically cooled CCD detector. Freshly prepared aliquots of MLV suspension (10 to 20 μL), following a freeze–thaw process as described in the Sample Preparation section, were deposited onto clean glass coverslips (#1.5). The remaining aqueous solvent was evaporated to create a solid-supported lipid bilayer film by placing the coverslips on a heating plate in a custom-made, sealed chamber at approximately 30 °C. Three independent samples were prepared, and for each sample, multiple scans (averaging 3 regions) were performed with 20 accumulations. A 40× microscope objective with a numerical aperture of 0.60 and a grating consisting of 1200 lines per millimeter were used for these measurements. All spectroscopic experiments were performed at ambient temperature.

## Results and Discussion

### Transbilayer Water Permeability

**Figure 2:**
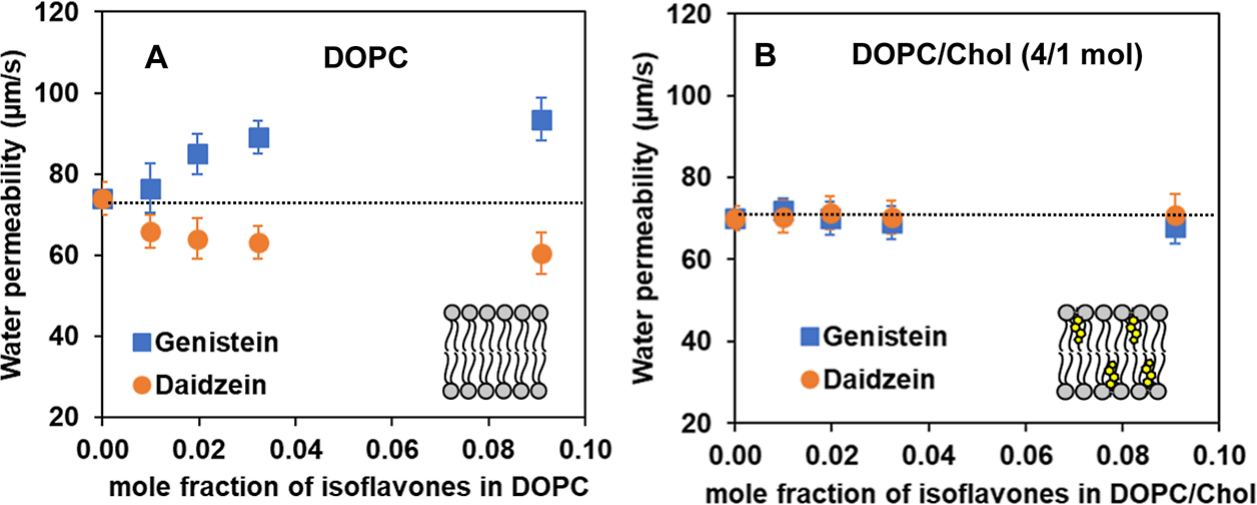
Transbilayer osmotic water permeability coefficients (μm/s) across lipid bilayers formed from (A) DOPC and (B) DOPC/Chol (4/1 mol ratio) at 30 °C in the presence of varying mole fractions of soy isoflavones (GEN, blue squares; DAI, orange circles). The horizontal dotted lines indicate the control values (74 μm/s for pure DOPC membranes and 70 μm/s for DOPC/Chol membranes without isoflavones).

Figure 2 shows the transbilayer osmotic water permeability (P_f_) of model membranes composed of DOPC lipids (or DOPC with Chol, 4 to 1 mol ratio) at 30 °C as a function of GEN and DAI mole fractions. The corresponding permeability coefficients are shown in Table S1 (Supporting Information). As seen in Figure 2A and Table S1, with an increasing concentration of GEN (blue squares), water permeability at 30 °C increases in the presence of 100:1, 50:1, and 30:1 DOPC to GEN mol ratios: from 74 μm/s (DOPC as a control) to 76, 85, and 89 μm/s, respectively. At a 10:1 DOPC to GEN mol ratio, the water permeability reaches 94 μm/s, which is an increase of about 27% relative to pure DOPC. On the other hand, the presence of DAI actually decreases the water permeability of the DOPC bilayer. With an increasing concentration of DAI (orange circles in Figure 2A), water permeability decreases to 66, 64, and 63 μm/s in the presence of 100:1, 50:1, and 30:1 DOPC to DAI mol ratios, respectively. At a 10:1 DOPC to DAI mol ratio, the water permeability reaches 60 μm/s, which is about 19% decrease from the water permeability of the bilayer formed from the pure DOPC bilayer under the same conditions. Overall, the magnitude of enhancement of water permeability of the DOPC bilayer induced by GEN is greater than that of reduction by DAI at the highest concentration (10:1 DOPC/isoflavones mol ratio).

We also explored water transport phenomena across cholesterol-containing DOPC bilayers to determine the role of this sterol lipid on bilayer water permeability in the presence of GEN and DAI. We employed cholesterol in a DOPC/Chol mol ratio of 4/1, a value at which the mixture reportedly does not exhibit phase separation.^60^ As shown in Figure 2B and Table S1, the presence of cholesterol at a 4/1 mol ratio of DOPC/Chol (without addition of any GEN or DAI) results in a reduction of the water permeability from 74 (pure DOPC) to 70 μm/s at 30 °C. This is consistent with cholesterol’s well-established condensing effect on the other lipidic components of the membrane, which in turn generally decreases area per lipid molecule and increases hydrocarbon thickness, both of which are correlated to a decrease in water permeability.^61^Figure 2B and Table S1 show the effects of varying concentrations of GEN and DAI on the water permeability values of this DOPC/Chol bilayer (4/1 mol ratio). The amount of isoflavones is denoted in terms of the mol ratio of total lipid (DOPC and Chol) to isoflavones, which was in the range from 100:1 to 10:1. As seen in Figure 2B and Table S1, both GEN and DAI exhibit almost negligible effects on water permeability on these cholesterol-containing bilayers within the standard deviation (SD) upon increasing concentrations of GEN or DAI. At best, there appears to be a slight decrease in water permeability from 70 to 68 μm/s at the highest GEN concentrations (10:1 lipid/GEN mol ratio). These results are in sharp contrast with the case of the pure DOPC bilayer, where the water permeability increased with increasing GEN concentrations. Similarly, there are no changes in water permeability as a function of DAI concentrations, whereas a gradual decrease of water permeability was observed in the situation for the pure DOPC bilayer. The foregoing results may be indicative of the ability of cholesterol to interfere with or obscure isoflavone-induced modulation of the bilayer physical properties that govern water permeability.

In general, the bilayer permeability of water has been known to depend on various structural and physical characteristics of individual lipids and the bilayers that they form. These factors include bilayer thickness, the area occupied by each molecule, polyunsaturation, and the overall fluidity of the membrane.^61−63^ The fluidity or rigidity of bilayers typically correlates with the packing density of lipids,^64^ and it is indeed expected that water permeability is influenced by the lipid packing within the bilayer region. Previous reports based on ATR-IR show that GEN (and other flavonoids) induces an increase of the number of gauche conformers in the hydrocarbon chains of a phosphocholine membrane, indicating the loosening of the bilayer structure.^27^ GEN’s ability to modulate gramicidin-A (gA) channel function, which is less pronounced for DAI, has been interpreted as an ability of GEN to alter bilayer mechanical properties and reduce the energy of hydrophobic mismatch of the gA channel to the lipid bilayer by making the bilayer softer and more easily deformable, which in turn modulates channel function.^23^ Furthermore, X-ray scattering studies and molecular dynamics (MD) simulations have revealed that both GEN and DAI, when introduced into DOPC and diphytanoyl-PC, affect the structural and elastic properties of lipid bilayers. These soy isoflavones are found to reduce membrane thickness, lower the bending modulus, and decrease the area compressibility modulus, collectively indicating a softening effect on the bilayers by both isoflavones. In fact, GEN appears to have a greater ability to thin the membrane and lower the area compressibility modulus than does DAI.^21^ Our findings which show that GEN increases transbilayer water permeability point to, in part, a possible increase in the fluidity of the bilayer.

Interestingly, our findings suggestive of increased bilayer fluidity consequent to interaction with GEN appear to contradict earlier reports indicating a membrane rigidifying capability of GEN (see summary described in the Introduction section in Table 1). Moreover, the opposing water permeability effects we observed, which were induced by GEN and DAI, signify a markedly unequal effect for bioactive flavonoid molecules depending on subtle differences in molecular structures. Among many factors that contribute to the modulation of the interaction of flavonoids with lipid membranes is the presence of different substituents in the backbone structure. Flavonoids are known to have a varying degree of hydroxylation,^65^ conferring a high capacity to form hydrogen bonds,^66^ which could indicate that H-bond formation between flavonoid molecules and the polar membrane interface markedly impacts their interactions.^52^ While GEN has one additional OH group compared to DAI, GEN is actually considered to be *more* hydrophobic than DAI due to its ability to form an intramolecular hydrogen bond: a phenolic −OH with its adjacent carbonyl group. This formation of intramolecular hydrogen bonding has been substantiated by ^1^H NMR line width experiments,^67^ and the formation of intramolecular hydrogen bonding has been computed to be effective 95% of the time by MD simulations.^21^ The imparted hydrophobicity is also evidenced by the octanol–water partition coefficient (log *P*) values of 3.04 and 2.51 for GEN and DAI, respectively.^68^ The hydrophobicity (log *P*) of a molecule has been correlated with the extent of its interaction with biological membranes.^68^ GEN, in addition to an interaction with the lipid headgroup, may also interact with the acyl chain region of the lipid bilayer environment, leading to perturbation of the acyl chain region and increased water permeability across the DOPC lipid bilayer. On the other hand, the less hydrophobic DAI is more limited to interaction with the lipid headgroup region, and the formation of hydrogen bonds with the polar membrane interface may dehydrate the polar headgroup, inducing membrane rigidity to thereby reduce transbilayer water permeability. Polyphenols such as flavonoids have an amphiphilic character that drives their interaction and penetration: their aromatic rings have hydrophobic character, and their phenolic hydroxyls can act as H-bond donors to the phospholipid headgroups. As a result, polyphenols should orient immediately below the membrane surface and, in this way, inhibit passive permeability. For DAI, this is consistent with a report demonstrating that DAI is located in the proximity of the membrane surface and rigidifies a soybean PC liposome.^32^ In the study presented here, we delivered equal molar quantities of GEN and DAI to the bilayer. Since GEN is more hydrophobic than DAI, it is likely that a greater relative quantity of GEN is partitioned into the bilayer. There is precedent for the differential effects of GEN *vs* DAI to be based on their relative hydrophobicity and membrane solubility when explaining the greater effect of GEN on gA channel functions as compared to that of DAI.^21^ A solubility limit for GEN and DAI in DOPC bilayers has been reported by X-ray data.^21^ While the isoflavonoid concentration ranges we employed were below the solubility limit, aggregation of phenolic flavonoid molecules is a known phenomenon as a result of Π-stacking and hydrogen bonding interactions.^69^

Relatively fewer reports have addressed the effect of GEN and DAI on cholesterol-containing model membranes. One such study reports that the extent of DAI-induced aggregation of liposomes is significantly reduced with the inclusion of 10 mol % cholesterol.^25^ In another study, fluorescence polarization indicated that GEN would rigidify the hydrophobic region of liposomal membranes with 20 mol % cholesterol and 80 mol % POPC, whereas DAI was not effective at the same concentrations.^30^ In that study, when the cholesterol concentration was increased to 40 mol %, the effect on the membrane was negligible or weaker compared to that of the membrane containing 20 mol % cholesterol.^30^ Our water permeability data showing a slight P_f_ decrease induced by GEN for the DOPC/Chol bilayer (4/1 mol ratio), but a negligible effect induced by DAI, is qualitatively consistent, considering that POPC (16:0–18:1 PC, *T*_m_ = −2 °C) is in a similarly fluidic phase as DOPC (18:1–18:1 PC, *T*_m_ = −17 °C) at ambient temperature.

It is evident that the presence of cholesterol strongly offsets both the membrane fluidizing effect of the GEN and the membrane rigidifying effect of the DAI when compared to the effect of these molecules on the pure DOPC bilayer. These results signal the important role of cholesterol in membrane stability and demonstrate how cholesterol-containing membranes appear to have an ability to resist global changes in their physical properties. We have previously demonstrated such a role of cholesterol relative to the effect of other bioactive molecules, where the membrane rigidifying or fluidifying effect was counteracted by increasing concentrations of cholesterol in lipid bilayers.^48,49^ The addition of cholesterol to the bilayers might be expected to alter the packing of the polar headgroups of the phospholipid molecules and change the water concentrations within the bilayer.^70^ This in turn may influence the hydrogen bonding capability between the hydroxyl moieties of isoflavones and the headgroups of lipid bilayers. In order to glean greater insight into the causes of the relative effects of GEN and DAI on the barrier properties of these model membranes, we next sought data from complementary studies through DSC and Raman spectroscopy.

### Thermotropic Properties

**Figure 3:**
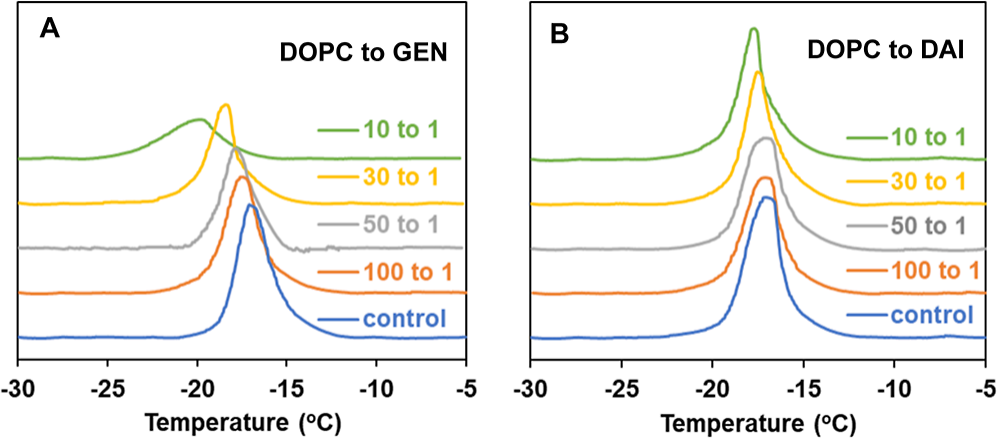
Endothermic calorimetric thermograms of DOPC MLVs containing different concentrations of (A) GEN and (B) DAI; the *Y*-axes of both (A,B) are scaled to be identical for comparison.

We investigated the influence of the studied isoflavones on the endotherms for the phase transition of MLVs composed of DOPC and DOPC/Chol by DSC. Figure 3 shows the endothermic thermograms of the phase transitions in DOPC MLVs in the presence of GEN and DAI, with the corresponding tabulated thermodynamic data shown in Table S2 (Supporting Information). The thermogram for the control DOPC MLVs (no isoflavones) in Figure 3 shows the well-defined endothermic transition of the lamellar gel phase L_β_ to the lamellar liquid-crystalline state L_α_ with a main phase transition temperature (*T*_m_) of around −17 °C and an associated enthalpy of the main transition (Δ*H*) of 8.2 to 8.8 kcal/mol. This is consistent with literature data (*T*_m_ = −18.3 ± 3.6 °C, Δ*H* = 9.0 ± 2.8 kcal/mol).^71^

On comparison of the thermograms, the dissimilar influence on the thermotropic phase behavior of DOPC MLVs is clearly apparent between GEN and DAI. As seen in Figure 3A, GEN has a significant effect on the thermogram of DOPC bilayers, with concentration-dependent changes evident in thermotropic phase behavior (also tabulated in Table S2). At a 100:1 mol ratio of DOPC to GEN, *T*_m_ is slightly decreased by ∼0.7 °C compared to that of the control, with about a 10% decrease in Δ*H* (from 8.76 at control to 7.80 kcal/mol). With increasing concentrations of GEN, *T*_m_ gradually further decreases to a lower temperature (*e.g.*, −18.81 °C at a 30:1 mol ratio compared to −17.08 °C at the control). At the same 30:1 mol ratio, Δ*H* was reduced by about 40% (4.91 kcal/mol compared to the control, 8.76 kcal/mol). When GEN is present at elevated concentration (10:1 mol ratio of DOPC to GEN), a marked peak broadening is observed (Δ*T*_1/2_, the width at half height of the main transition, of 3.32 °C *vs* 2.04 °C at the control), indicating disruption of lipid packing and the reduction in transition cooperativity in the lipid bilayer environment, possibly arising from the existence of a heterogeneous phase with an uneven GEN distribution. On the contrary, in the case of DAI, the DOPC thermogram is overall minimally affected (Figure 3B) compared to that of GEN. As shown in Figure 3B, increasing concentrations of DAI lead to a slight shift toward lower *T*_m_ and negligible reductions in Δ*H*, with little or no peak broadening of the main phase transition, suggestive of a lack of significant disruption of the acyl chain environments.

**Figure 4:**
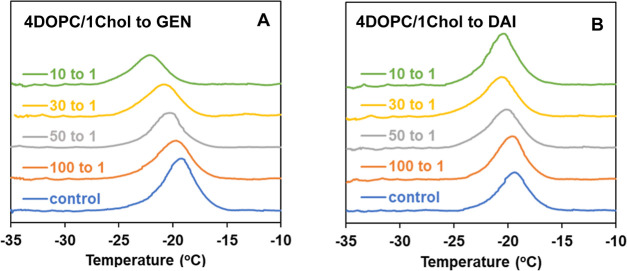
Endothermic calorimetric thermograms of DOPC/Chol (4/1 mol/mol) MLVs containing different concentrations of (A) GEN and (B) DAI; the *Y*-axis is scaled to be identical for (A,B) for comparison.

Figure 4 shows the endothermic thermograms of DOPC/Chol MLVs (4/1 DOPC/Chol mol ratio) and their modification in the presence of GEN and DAI at different concentrations. The addition of Chol to DOPC is well-known to affect its thermogram in both *T*_m_ and Δ*H*, as was the case in our results shown in the control sample (DOPC/Chol at a 4/1 mol ratio without any isoflavones) in Figure 4 (blue traces). Our results, showing a decrease of *T*_m_ of about 2 to 2.5 °C with a nearly 60% reduction in Δ*H* and overall broadening (Δ*T*_1/2_) compared to that of pure DOPC, are consistent with previous reports on cholesterol-containing DOPC bilayers.^72^ As the mole fraction of GEN in DOPC/Chol increases, the main phase transition peak is moved toward a lower temperature with a gradual reduction of the enthalpy of transition (Δ*H*), as seen in Figure 4A and Table S3. In contrast, with increasing concentrations of DAI, *T*_m_ and Δ*H* are minimally affected, as shown in Figure 4B and Table S3.

**Figure 5:**
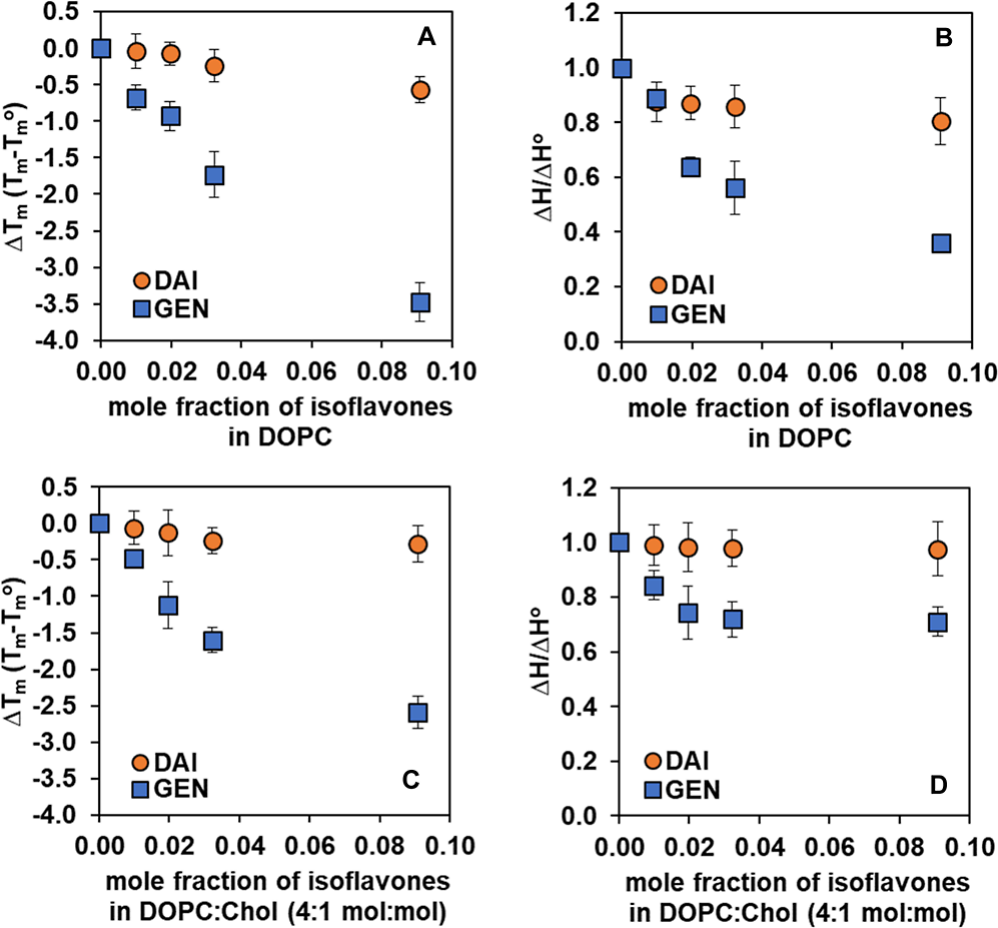
Relative comparison of the effect of GEN (blue squares) and DAI (orange circles) on the thermodynamic parameters of (A,B) DOPC and (C,D) DOPC/Chol (4/1 mol/mol) MLVs.

For relative comparison, Figure 5A,C shows the changes in main phase transition temperature (Δ*T*_m_ = *T*_m_ – *T*_m_°), where *T*_m_ is the main phase transition temperature in the presence of given concentrations of isoflavones and *T*_m_° is the main phase transition temperature in the absence of any isoflavones, plotted as a function of mole fraction of isoflavones for DOPC (Figure 5A) and DOPC/Chol (Figure 5C). Analogously, Figure 5B,D plots the changes of the enthalpy of transition (shown as the ratio Δ*H*/Δ*H*°) as a function of the mole fraction of isoflavones for DOPC (Figure 5B) and DOPC/Chol (Figure 5D), where Δ*H*° is the transition enthalpy in the absence of isoflavone. As seen in Figure 5A–D, GEN induces more profound changes in Δ*T*_m_ and Δ*H*/Δ*H*° than its structurally similar soy isoflavone DAI for both DOPC and DOPC/Chol membranes.

Our data, showing that the presence of GEN induces a marked decrease in the change in phase transition temperature (Δ*T*_m_) and reduction in Δ*H*/Δ*H*° for DOPC membranes (blue squares in Figure 5A,B), indicate that GEN can penetrate into the acyl chain of DOPC, induce molecular disordering, and lead to destabilization and greater fluidization of the bilayer membrane. In comparison, DAI induced a significantly lessened extent of Δ*T*_m_ lowering, as well as a relatively smaller reduction in Δ*H*/Δ*H*° of DOPC lipid membranes (orange circles in Figure 5A,B). The observation of there being no significant changes in Δ*H*/Δ*H*° indicates the maintenance of an orderly arrangement within the acyl chain core of DOPC. It may be hypothesized that DAI has a predominant population at the bilayer surface to interact *via* hydrogen bonding with the polar headgroups of the lipid, thus only minimally affecting *T*_m_ and Δ*H*. In comparison, GEN has an ability for relatively greater penetration into the acyl chain region. An analogous set of differential effects are seen in the lipid membranes of DOPC/Chol: the extent of decrease of Δ*T*_m_ is greater for GEN (blue squares in Figure 5C) than that for DAI (orange circles in Figure 5C) at all concentrations, as was the case for pure DOPC. This may signal that hydrophobic GEN may partition into the lipid bilayer even in the presence of cholesterol, as evidenced by the reduction of Δ*H*/Δ*H*° (Figure 5D), albeit to a somewhat lesser extent compared to that of pure DOPC. In contrast, DAI may be excluded from the hydrocarbon core to position at the interface of lipid membranes, minimally influencing transition temperature and enthalpic changes. Overall, the thermal phase behavior of GEN and DAI shows markedly different effects, apparently consistent with the water permeability results described in the previous section.

There have been prior DSC studies showing the perturbation of the thermal phase behavior of model membranes by soy isoflavones. Both DAI and GEN have been reported to reduce the Δ*H* and pretransition temperature of DMPC and DPPC in a concentration-dependent manner.^25^ On the other hand, opposite results have been reported as well, where GEN has been reported to induce an increase in the *T*_m_ value and Δ*H* of DMPC.^28^ To the best of our knowledge, there are no DSC data addressing or observing differing interactions of GEN and DAI with DOPC and DOPC/Chol lipid membranes; hence, no direct comparison to our present data is available. When the phospholipid molecules are relatively loosely packed, as in the DOPC bilayer, the lipid bilayer environment would have sufficient initial flexibility to accommodate the partitioning of a hydrophobic molecule, such as GEN, into the bilayer. Therefore, it is possible that the nature and extent of interaction are dependent upon the lipid composition, particularly between the fluid membrane consisting of unsaturated acyl chains, such as DOPC, as used in our study, and the saturated acyl chains, such as DPPC and DMPC, studied by others.

### Structural Property

**Figure 6:**
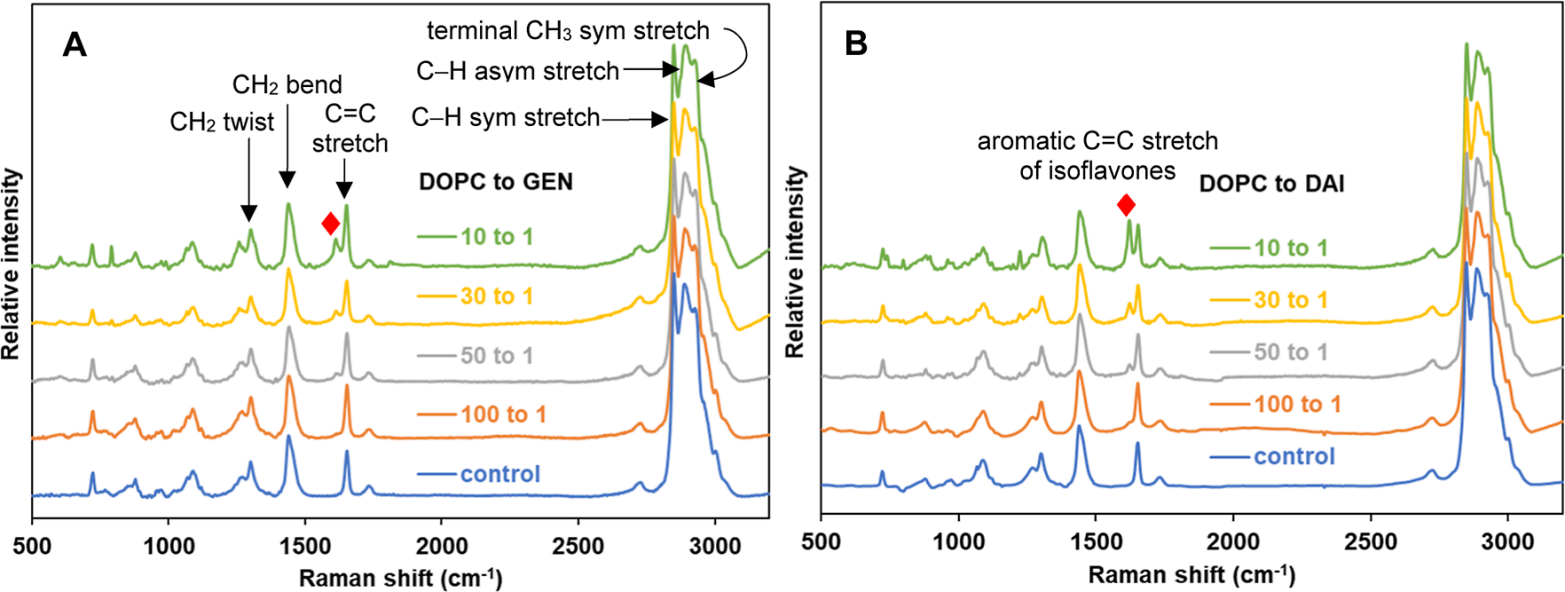
Raman spectra of DOPC with increasing concentrations of (A) GEN and (B) DAI at room temperature.

Figure 6 shows the room-temperature Raman spectra of supported lipid bilayers of DOPC containing varying concentrations of GEN and DAI. All spectra are baseline corrected and normalized to the intensity at ∼2848 cm^–1^ for comparison. The characteristic Raman bands of the DOPC bilayer include the following: CH_2_ twist and bend (∼1300 and ∼1440 cm^–1^), C=C stretching (∼1650 cm^–1^), and C–H stretching (∼2800–3100 cm^–1^). For isoflavone-containing bilayers, there is an increasing intensity of the peak at ∼1614 cm^–1^, which is attributed to an aromatic C=C stretching from GEN or DAI (marked as red diamond), with increased concentrations of these molecules.^73^ The Raman spectra of GEN and DAI (Figure S1), along with detailed peak assignments for DOPC and these isoflavone molecules, are shown in Table S4 in the Supporting Information.

**Figure 7:**
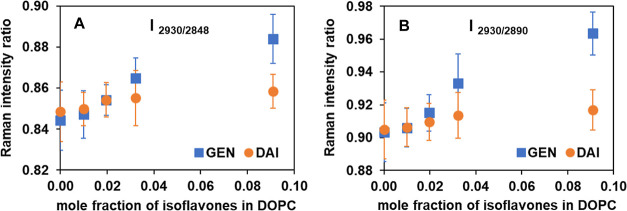
Raman peak intensity ratios **I** of (A) [C–H_term_ (2930)/C–H_sym_ (2848)] and (B) [C–H_term_ (2930)/C–H_asym_ (2890)] of DOPC with increasing concentrations of GEN (blue square) and DAI (orange circle) at room temperature. Each data point represents the average and SD derived from three independently prepared samples, and each sample is scanned across three different sample regions.

Raman spectra for supported phospholipid bilayers have been extensively studied, especially in the C–H stretching region (2700–3100 cm^–1^), due to the strong Raman scattering characteristics in this section. This region is known to provide insights into the order of the hydrocarbon chains. This region consists of the methylene C–H symmetric and asymmetric stretching modes at ∼2848 and 2890 cm^–1^, respectively, and the terminal methyl C–H (of the hydrocarbon chain) symmetric stretching mode at ∼2930 cm^–1^.^74,75^ Note that there are no interferences from Raman bands originating from GEN and DAI in this region (Figure S1 and Table S4). The use of these specific peaks and their corresponding intensities in the C–H stretching regions is well-established for assessing various membrane structural properties. These properties include chain decoupling, rotational disorder, relative acyl chain order/disorder parameters, and packing effects.^74−76^ Consequently, any structural alterations in the DOPC membranes resulting from their interaction with GEN and DAI are tracked by examining spectral differences within this region. Figure 7 shows the ratios of peak intensities of **I**, specifically [C–H_term_ (2930)/C–H_sym_ (2848)] and [C–H_term_ (2930)/C–H_asym_ (2890)], which serve as indicators of acyl chain packing within the membrane. Specifically, an increase in the I_2930/2848_ [C–H_term_/C–H_sym_] intensity ratio indicates an increase in rotational disorder and freedom of motion, while an increase in the I_2930/2890_ [C–H_term_/C–H_asym_] ratio infers a decrease in both intramolecular (gauche/trans) and intermolecular (chain packing) interactions. The corresponding tabulated values are also shown in the Supporting Information (Table S5). Figure S2A shows the comparison of the Raman spectra in the C–H stretching region (2700–3100 cm^–1^) for GEN and DAI at a 10 to 1 molar ratio of DOPC to these isoflavone molecules.

Figure 7A shows that an increased concentration of GEN in DOPC-supported bilayers results in a significant increase in the peak intensity ratio of [C–H_term_ (2930)/C–H_sym_ (2848)] from 0.84 to 0.88, while there is a relatively negligible change shown for DAI at the same concentration (10 to 1 mol ratio of DOPC to isoflavone). Similarly, for the peak intensity ratio of [C–H_term_ (2930)/C–H_asym_ (2890)], the ratios are observed to change significantly from 0.90 to 0.96 for GEN, whereas little change, from 0.91 to 0.92, is seen for DAI, as shown in Figure 7B. Our Raman results indicate that these ratios increase with progressively greater concentrations of GEN molecules interacting with a DOPC bilayer. This suggests that there is a weakening of intermolecular interactions between acyl chains due to the greater presence of soy isoflavone molecules, which disrupts the packing order. This effect is more pronounced in the case of GEN compared to that of DAI.

The acyl chain decouples more pronouncedly with increased concentrations of isoflavone and results in increased rotational and vibrational freedom of the terminal methyl group, resulting in increased ratios of [C–H_term_ (2930)/C–H_sym_ (2848)] and [C–H_term_ (2930)/C–H_asym_ (2890)]. Our findings from these Raman spectroscopic studies are consistent with the increasing water permeability for increased GEN concentrations: the greater the degree of disorder and decreased packing density, the greater the water permeability. On the other hand, DAI did not induce any significant hydrocarbon chain disordering effect, consistent with no increase in water permeability (instead, a modest decrease in water permeability, as described earlier). These are also consistent with our DSC results, which show a more pronounced decrease in *T*_m_ and a marked reduction of Δ*H* in the presence of GEN compared to the presence of DAI.

**Figure 8:**
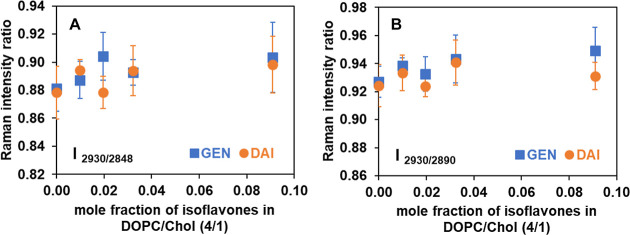
Raman peak intensity ratios **I** of (A) [C–H_term_ (2930)/C–H_sym_ (2848)] and (B) [C–H_term_ (2930)/C–H_asym_ (2890)] of DOPC/Chol (4/1, mol/mol) with increasing concentrations of GEN (blue square) and DAI (orange circle) at room temperature. Each data point represents the average and SD derived from three independently prepared samples, and each sample is scanned across three different sample regions.

We conducted similar Raman spectroscopic studies on the DOPC/Chol membrane system to investigate its interaction with GEN and DAI. The Raman intensity ratios **I** in the C–H stretching region are presented in Figure 8, and the corresponding ratios are tabulated in Table S6 of the Supporting Information. Figure S2B shows the comparison of the Raman spectra in the C–H stretching region (2700–3100 cm^–1^) for GEN and DAI at a 10 to 1 molar ratio of DOPC/Chol (4/1 mol/mol) to these isoflavone molecules.

As seen in Figure 8 and Table S6, the Raman peak intensity ratios overall evince a lesser extent of changes for both GEN and DAI. An increasing concentration of GEN and DAI in DOPC/Chol-supported bilayers results in correspondingly negligible changes in the peak intensity ratio of [C–H_term_ (2930)/C–H_sym_ (2848)] at the highest concentration, a 10 to 1 mol ratio of DOPC to isoflavone molecules. For the peak intensity ratio of [C–H_term_ (2930)/C–H_asym_ (2890)], the ratios are observed to change from 0.93 to 0.95 for GEN and from 0.92 to 0.93 for DAI, again indicating no significant differences between GEN and DAI in the presence of cholesterol. In summary, our findings align with the hypothesis that the hydrophobic nature of GEN allows it to penetrate deeper into the lipid bilayer, leading to disruption of the lipid packing. In contrast, the structure of DOPC/Chol remains largely undisturbed by DAI, indicating its probable location at the surface of the bilayer membranes.

## Conclusions

An increased awareness of health among the public has brought significant attention to the benefits of naturally occurring dietary supplements. Among these supplements, the two major soy isoflavones, GEN and DAI, are phytochemical polyphenolic compounds with potential health advantages. Interactions between bioactive molecules and cell membranes can modify the biophysical properties of the latter, and such perturbations often profoundly affect protein-mediated functions. Nevertheless, it is generally not well understood how bioactive molecules impact the physical and structural properties of cell membranes, including dynamic properties, fluidity, and lipid membrane packing.

In this study, we examined the interaction of GEN and DAI with model bilayer membranes of two different lipid compositions: DOPC and DOPC with cholesterol (4 to 1 mol ratio). We employed a suite of techniques designed to investigate several aspects: (1) transbilayer water permeability across the DIB to deduce modulations in the bilayer’s physical state; (2) phase transition behavior of MLVs to comprehend changes in membrane fluidity using DSC; and (3) membrane structural properties, such as relative acyl chain order/disorder parameters and packing effects, using confocal Raman microspectroscopy.

The combined results from these complementary experimental techniques provide evidence of nonspecific interactions between GEN and DAI and model lipid membranes. Although the two soy isoflavone molecules examined in this study share structural similarities, the nature and extent of their nonspecific interactions with model membranes greatly depend on subtle differences in their molecular structures, concentrations of isoflavone molecules, and lipid composition, particularly the presence of cholesterol, as summarized in Table 3.

**Table 3:**
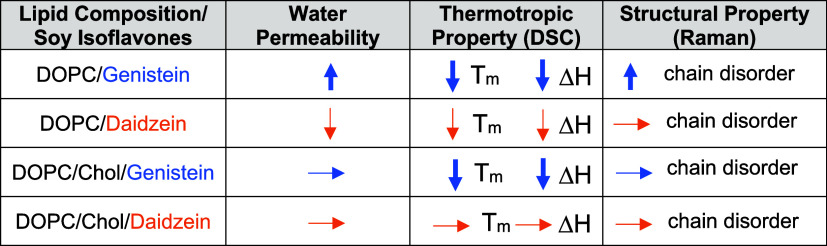
Summary of Distinct Impacts of GEN and DAI with Model Lipid Membranes of DOPC and DOPC with Cholesterol (4:1 Molar Ratio) Composition^a^

a Direction of the arrow indicates the increase (up), decrease (down), and negligible change (sideways) with its thickness representing the strength of interactions (stronger: thick; weaker: thin).

As shown in Table 3, our data show contrasting effects of GEN and DAI on the water permeability of DOPC membranes under the same conditions. GEN induces an increase in water permeability, while DAI brings about a decrease. In the presence of cholesterol in the DOPC membrane (at a DOPC-to-cholesterol molar ratio of 4 to 1), a less pronounced and varied influence on water permeability is observed between GEN and DAI. The phase transition behavior of DOPC membranes in the presence of GEN or DAI, at increasing concentrations, also reveals differences in the modification of membrane fluidity. This is reflected by a decrease in *T*_m_ and a reduction in Δ*H*, with GEN having a markedly greater effect on the thermotropic properties compared to that of DAI. The presence of cholesterol in DOPC also creates a system in which these respective isoflavone molecules exhibit disparate effects: GEN continues to lower *T*_m_ and decrease Δ*H*, whereas DAI shows no significant changes in *T*_m_ and Δ*H*. Raman spectroscopic studies indicate that the extent of chain disordering is more pronounced in DOPC with GEN compared to that with DAI. The substantial disruption of the phase transition behavior and acyl chain disorder induced by the presence of GEN in DOPC likely play a role in the increased water permeability observed in our system. On the other hand, the DAI-associated decrease in water permeability corresponds to the negligible changes in phase transition behavior and acyl chain disorder, indicating that DAI may be located at the membrane/water interface without significantly influencing the lipid bilayer structure, possibly hindering the passage of water transport. Collectively, our findings from various experimental methods, including water permeability, phase transition behavior, and vibrational spectroscopy for acyl chain disorder, are qualitatively consistent and suggest a set of significantly different interactions of GEN and DAI with model membranes of DOPC and DOPC containing cholesterol. Our experimental findings are also in good agreement with the previous work investigating the effects of GEN and DAI on the structural and elastic properties of model membranes by X-ray scattering and MD simulations.^21^ The findings of the latter showed a greater thinning of DOPC membranes with GEN than with DAI as well as a greater capability of GEN to reduce area compressibility and suggest that the partitioning of GEN into the membrane is greater than for DAI. Their electron density profiles also indicated that, with increasing concentrations, GEN is moved toward the bilayer center while DAI is moved outward from the bilayer center.^21^

The activity of flavonoid compounds has been correlated with their position within the lipid membrane because the presence of bioactive molecules embedded in the membrane can alter the order and packing of the acyl chain region of the lipid bilayer.^19^ This modification affects the temperature and enthalpy of the main phase transition and changes the fluidity of the membranes. The extent of this effect also depends on the molecular hydrophobicity. Both GEN and DAI have the ability to engage in hydrogen-bonding interactions through their hydroxyl groups with the polar headgroup of DOPC molecules and hydrophobic interactions between their phenyl rings and the acyl chains of the DOPC lipid bilayer. However, due to its higher hydrophobicity, GEN can more easily intercalate into the lipid membranes and penetrate the hydrophobic layer of the DOPC membrane, especially at high concentrations. It fluidizes the bilayer compared to DAI. Therefore, it is likely that DAI is primarily limited to the headgroup region and has less extensive acyl chain penetration, leading to a putatively predominant location at the membrane interface.

In the presence of cholesterol in the DOPC membrane, however, there is little change observed in water permeability and acyl chain disordering, as well as a relatively minimal extent of changes in *T*_m_ and Δ*H*. It would seem that cholesterol’s widely recognized ability to increase chain rigidity and decrease membrane permeability exerts a counteracting effect on GEN’s ability to increase water permeability or DAI’s ability to decrease it in the DOPC bilayer. This may indicate that either cholesterol is competing with these molecules at a site of membrane interaction or that the changes induced in the membrane by the presence of cholesterol hinder the interaction of these molecules with the membrane. It is possible that there is competition between GEN and cholesterol in the hydrophobic core, and the partitioning of GEN to the extent observed in pure DOPC membranes is inhibited, likely due to a relative lack of free volume in the DOPC/cholesterol system. It has been reported that the maximum solubility of GEN in DOPC vesicles decreases with an increasing composition of cholesterol, with about a 20% decrease in the presence of 30% cholesterol.^77^ In the cholesterol-free DOPC system, GEN can interact with both the headgroups and the acyl chain region of lipid bilayers. However, in the presence of cholesterol, which is well-known to reside closer to the membrane hydrophobic core, it is likely that GEN is limited to positioning itself closer to the headgroups through hydrogen bonding interactions with the phosphate and ester groups of lipids.

In the case of DAI, its relatively lower hydrophobicity (and therefore less solubility in the membrane core) limits its position to the interface of water/lipid membranes, consistent with the reported MD simulations.^21^ As a result, the effect of cholesterol in the membrane will likely have a relatively less significant impact on the overall interactions of DAI with the DOPC membrane compared to that in the case of GEN. However, it is possible that DAI’s ability to position at the membrane interface is inhibited due to cholesterol-induced modifications in the polar headgroup environment. Competition between DAI and cholesterol, both anchored at the interface, might disrupt the orientation of cholesterol molecules and compromise cholesterol’s reputed ability to impose order. This is consistent with the observation that there were no significant changes in water permeability in the presence of cholesterol.

Our results underscore the significant interplay each solute has with lipid bilayers, and their effects are highly dependent on the molecular structure and hydrophobicity of these bioactive isoflavone molecules as well as the presence of cholesterol in the membrane. These effects provide valuable insights into the mechanisms of interaction between bioactive molecules and cell membranes in a heterogeneous environment, which can contribute to a better understanding of relevant physiological processes and pharmaceutical applications.

## Supporting Information Available

Determination of water permeability using the DIB model membrane; water permeability coefficient for DOPC and DOPC/Chol (4/1 mol/mol) membranes as a function of isoflavone concentrations; thermodynamic parameters for DOPC and DOPC/Chol MLVs at different concentrations of GEN and DAI; Raman spectra of GEN and DAI; selected characteristic peak assignments of DOPC and isoflavones Raman spectra; and Raman intensity ratios of DOPC and DOPC/Chol with isoflavone concentrations (PDF)
