# Supplementary material for: Differential Effects of Soy Isoflavones on the Biophysical Properties of Model Membranes
Source: J Phys Chem B. 2024 Feb 28;128(10):2412–24. doi: 10.1021/acs.jpcb.3c08390 (PMC10945484; doi:10.1021/acs.jpcb.3c08390)
Supplement: Supplementary file 1 — jp3c08390_si_001.pdf [file jp3c08390_si_001.pdf]

Supporting Information

Differential effects of soy isoflavones on biophysical properties of model membranes

*Jamie Gudyka, Jasmin Ceja-Vega, Katherine Ivanchenko, Wilber Perla, Christopher Poust, Alondra Gamez Hernandez, Colleen Clarke, Shakinah Silverberg, Escarlin Perez, and Sunghee Lee**

Department of Chemistry and Biochemistry, Iona University, 715 North Avenue, New Rochelle, New York 10801, USA

*To whom correspondence should be addressed. Tel: 914-633-2638. Fax: 914-633-2240.
E-mail: SLee@iona.edu

Determination of water permeability using DIB model membrane

The water permeability measurement was performed using the model membrane formed by the droplet interface bilayer (DIB) method. A DIB is formed when aqueous microdroplets bounded by lipid monolayers create a region that has a structure essentially the same as the double-leaflet lipid bilayer of cell membranes (Figure 1 in main article). When two osmotically unbalanced microdroplets were made to adhere at a bilayer, the osmotic gradient drives water transport through the droplet bilayer (the direction of water transport is shown with the arrow in Figure 1), resulting in a visible change in droplet diameter. Any electrolyte flux is expected to be negligible compared to that of water, as ion permeation is typically almost eight orders of magnitude slower than that of water. The corresponding changes in droplet volume over time (dV/dt) is measured optically by microscopic observation; and the behavior of the system follows the expression of equation (1) based on Fick's Law:

$$\frac{\mathrm{d}V(t)}{\mathrm{d}t} = -P_f A(t) \nu_w \Delta C(t) \qquad (1)$$

where A is the geometric bilayer area, ν_w is the molar volume of water (18 mL/mol), ΔC(t) is the osmolality gradient between two droplets, and P_f is the bilayer permeability coefficient of water. The volume change with time (dV/dt) is related to the bilayer permeability coefficient of water, P_f, as expressed in the Equation (1). When the bilayer contact area is constant, the time evolution of the swelling droplet can be obtained from the following equation derived from the integration

of eqn. 1, with the following simplifying assumption: since one of the droplets (the shrinking droplet) contains no osmotic agent, its concentration does not change with time:[1, 2]

$$\left(\frac{V}{V_o}\right)^2 = \left(\frac{2P_f A v_w C_o}{V_o}\right) t + 1 \qquad (2)$$

Using the measured values for: initial size of the osmotic (swelling) droplet; bilayer contact area (A); and initial osmolarity of the osmotic droplet (C_o), then the coefficient P_f for bilayer water permeability may be derived from eqn. 2 from the slope of the curve obtained by plotting $(V/V_o)^2$ as a function of time. All data points presented in this paper are an average ($n \geq 30$) of individual permeability runs, each of which took place over a time course (~5 min) for osmotic water movement across the droplet bilayer, during which time the droplet contact area (A) remains constant. The recorded videos and images were post-analyzed to measure the dimension of droplets and contact area using custom built image analysis software. All droplet pairs had substantially the same initial size relative to each other, in the diameter range of 100 ± 5 µm diameter.

Transbilayer Water Permeability

Table S1. Water permeability coefficient at 30°C for DOPC and DOPC/Chol (4/1 mol/mol) membrane, as a function of isoflavone concentrations.

Concentrations of isoflavone molecules	DOPC		DOPC to Chol (4 to 1 mole ratio)	
	Genistein	Daidzein	Genistein	Daidzein
control	74 ± 4	74 ± 4	70 ± 3	70 ± 3
100 to 1	76 ± 6	66 ± 4	72 ± 4	70 ± 3
50 to 1	85 ± 5	64 ± 5	70 ± 4	71 ± 4
30 to 1	89 ± 4	63 ± 4	69 ± 4	70 ± 4
10 to 1	94 ± 5	60 ± 5	68 ± 5	70 ± 4

[1] Lopez, M.; Evangelista, S. E.; Morales, M.; Lee, S. Enthalpic effects of chain length and unsaturation on water permeability across droplet bilayers of homologous monoglycerides. *Langmuir* 2017, 33 (4), 900-912.

[2] Thiam, A. R.; Bremond, N.; Bibette, J. From stability to permeability of adhesive emulsion bilayers. *Langmuir* 2012, 28 (15), 6291-6298.

Thermotropic Property

Table S2. Thermodynamic parameters (T_m, ΔH, Δ $T_{1/2}$) for main phase transition of DOPC MLVs at different concentration of GEN and DAI.

DOPC: Isoflavones (mol:mol)	GEN			DAI		
	T_m (°C)	ΔH (kcal/mol)	Δ $T_{1/2}$ (°C)	T_m (°C)	ΔH (kcal/mol)	Δ $T_{1/2}$ (°C)
1:0	–17.08 ± 0.13	8.76 ± 0.24	2.04	–17.11 ± 0.10	8.23 ± 0.35	2.04
100:1	–17.76 ± 0.11	7.80 ± 0.11	2.11	–17.15 ± 0.21	7.22 ± 0.44	2.18
50:1	–18.01 ± 0.15	5.59 ± 0.28	1.95	–17.18 ± 0.12	7.17 ± 0.32	2.23
30:1	–18.81 ± 0.28	4.91 ± 0.83	1.99	–17.35 ± 0.20	7.06 ± 0.51	2.00
10:1	–20.56 ± 0.23	3.16 ± 0.20	3.32	–17.68 ± 0.15	6.63 ± 0.60	1.97

Table S3. Thermodynamic parameters (T_m, ΔH, Δ $T_{1/2}$) for main phase transition of DOPC:Chol (4:1) MLVs at different concentration of GEN and DAI.

[4DOPC:1Chol]: Isoflavones (mol:mol)	GEN			DAI		
	T_m (°C)	ΔH (kcal/mol)	Δ $T_{1/2}$ (°C)	T_m (°C)	ΔH (kcal/mol)	Δ $T_{1/2}$ (°C)
1:0	–19.18 ± 0.10	3.13 ± 0.19	3.19	–19.33 ± 0.10	2.84 ± 0.13	2.89
100:1	–19.66 ± 0.05	2.64 ± 0.05	3.40	–19.39 ± 0.20	2.81 ± 0.17	2.90
50:1	–20.30 ± 0.30	2.33 ± 0.27	2.97	–19.46 ± 0.30	2.79 ± 0.22	3.29
30:1	–20.78 ± 0.14	2.25 ± 0.15	3.36	–19.57 ± 0.15	2.78 ± 0.14	3.43
10:1	–21.77 ± 0.19	2.22 ± 0.10	3.25	–19.61 ± 0.23	2.77 ± 0.25	3.04

Raman Spectroscopy

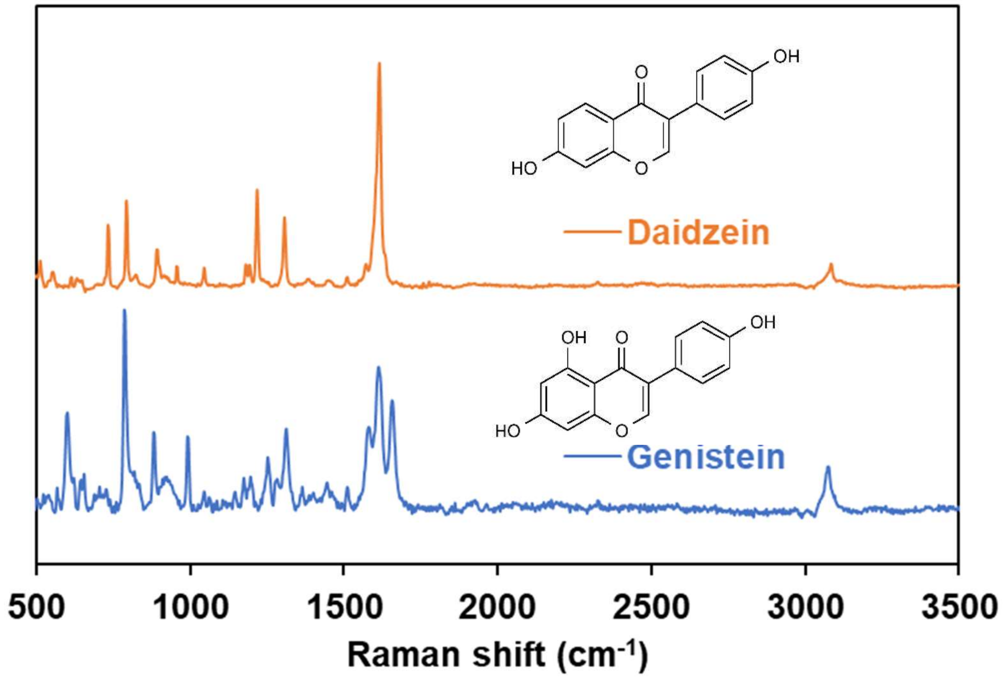

Figure S1. Raman spectra of genistein and daidzein (film) at room temperature.

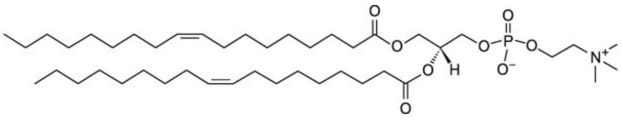

1,2-dioleoyl-*sn*-glycero-3-phosphocholine (18:1 (Δ9 cis) PC, DOPC)

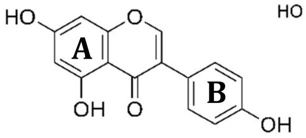

Genistein (GEN)

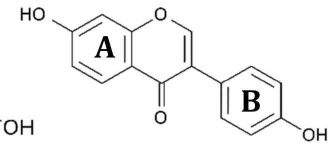

Daidzein (DAI)

Table S4. Selected characteristic peak assignments of DOPC and isoflavones Raman spectra

Raman band	DOPC*	Isoflavone molecules**
ν (choline CH_3 asymmetric)	3034 cm^{-1}	
ν (C−H)		3100-3000 cm^{-1}
ν (unsaturated C−H)	3004 cm^{-1}	
ν (CH_3 symmetric)	2928 cm^{-1}	
ν (CH_2 asymmetric)	2891 cm^{-1}	
ν (CH_2 symmetric)	2849 cm^{-1}	
ν (C=O)	1735 cm^{-1}	
ν (C=O) of ring B		1649-1694 cm^{-1}
ν (C=C)	1655 cm^{-1}	
ν (C=C) of ring		1623-1608 cm^{-1}
δ (CH_2)	1438 cm^{-1}	
τ (CH_2)	1299 cm^{-1}	
ν (C=O) + ν (C=C)		1200-1160 cm^{-1}
ν (C=C)		1185-1179 cm^{-1}
ν (C–C gauche)	1085 cm^{-1}	
ν (C–C trans)	1066 cm^{-1}	
ν (C=C)		991-970 cm^{-1}
δ (C-C-C) ring C + ν (C=C) ring A		890-880 cm^{-1}
ν (N-CH_3 symmetric)	872 cm^{-1}	
ν (N-CH_3 symmetric)	717 cm^{-1}	
δ (C-C-C) of ring B + δ (C–O)		566-547 cm^{-1}

* Ceja-Vega, J.; Perez, E.; Scollan, P.; Rosario, J.; Gamez Hernandez, A.; Ivanchenko, K.; Gudyka, J.; Lee, S. Trans-Resveratrol Decreases Membrane Water Permeability: A Study of Cholesterol-Dependent Interactions. The Journal of Membrane Biology 2022, 255 (4-5), 575-590;
Cherney, D.P.; Conboy, J.C.;Harris, J.M. Optical-trapping Raman microscopy detection of single unilamellar lipid vesicles. Analytical chemistry, 2003, 75(2 3), 6621-6628.
** Krysa, M.; Szymańska-Chargot, M.; Zdunek, A. FT-IR and FT-Raman fingerprints of flavonoids–a review. Food Chemistry 2022, 133430.

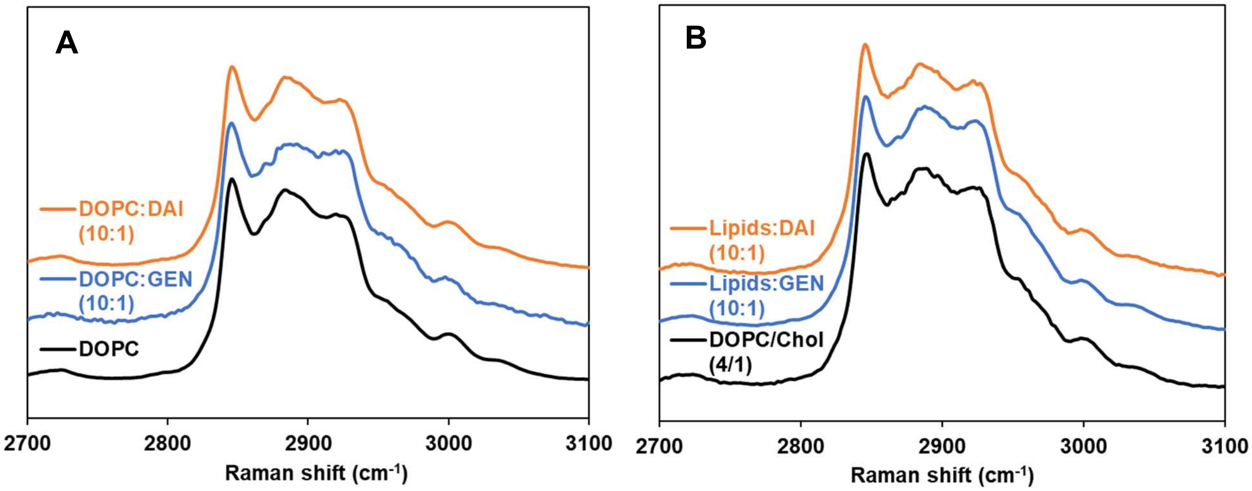

Figure S2. A comparison of the Raman spectra in the C–H stretching region (2700-3100 cm^{-1}) for GEN and DAI at 10 to 1 molar ratio of (A) DOPC and (B) DOPC/Chol (4/1 mol/mol) to these isoflavone molecules.

Table S5. Raman intensity ratios of [$C{-}H_{sym}$ (2848)/$C{-}H_{asym}$ (2890)] and [$C{-}H_{term}$ (2930)/$C{-}H_{asym}$ (2890)] of DOPC at ambient temperature. The corresponding plots are shown in the main article (Figure 7).

DOPC:isoflavone (mol:mol)	Raman intensity ratio			
	Genistein		Daidzein	
	I=2930/2848	I=2930/2890	I=2930/2848	I=2930/2890
1:0	0.844 ± 0.015	0.903 ± 0.018	0.848 ± 0.015	0.905 ± 0.018
100:1	0.847 ± 0.012	0.906 ± 0.012	0.850 ± 0.008	0.906 ± 0.012
50:1	0.854 ± 0.008	0.915 ± 0.011	0.854 ± 0.008	0.910 ± 0.011
30:1	0.865 ± 0.010	0.933 ± 0.018	0.855 ± 0.008	0.914 ± 0.014
10:1	0.884 ± 0.012	0.963 ± 0.013	0.858 ± 0.010	0.917 ± 0.012

Table S6. Raman intensity ratios of [$C{-}H_{sym}$ (2848)/$C{-}H_{asym}$ (2890)] and [$C{-}H_{term}$ (2930)/$C{-}H_{asym}$ (2890)] of DOPC/Chol (4/1) at ambient temperature. The corresponding plots are shown in the main article (Figure 8).

DOPC:isoflavone (mol:mol)	Raman intensity ratio			
	Genistein		Daidzein	
	I=2930/2848	I=2930/2890	I=2930/2848	I=2930/2890
1:0	0.881 ± 0.016	0.927 ± 0.011	0.878 ± 0.019	0.924 ± 0.015
100:1	0.887 ± 0.013	0.938 ± 0.006	0.894 ± 0.007	0.933 ± 0.013
50:1	0.904 ± 0.017	0.933 ± 0.012	0.878 ± 0.012	0.924 ± 0.008
30:1	0.893 ± 0.009	0.943 ± 0.017	0.894 ± 0.018	0.941 ± 0.016
10:1	0.904 ± 0.025	0.949 ± 0.017	0.898 ± 0.020	0.931 ± 0.010
